# Gastrointestinal Disease: New Diagnostic and Therapeutic Approaches

**DOI:** 10.3390/biomedicines11051420

**Published:** 2023-05-11

**Authors:** Beata Jabłońska, Sławomir Mrowiec

**Affiliations:** Department of Digestive Tract Surgery, Medical University of Silesia, 40-752 Katowice, Poland; mrowasm@poczta.onet.pl

Gastrointestinal diseases (GIDs) involve various benign and malignant pathologies of the digestive tract, as well as the liver, biliary tract, and pancreas [1]. The common clinical symptoms of GIDs are as follows: abdominal pain, heartburn, nausea, vomiting, bloating, diarrhea, constipation, and gastrointestinal bleeding [1].

In general, GIDs are divided into two main groups: functional and structural [1]. In the case of functional GID, the structure of the alimentary tract looks normal, but motility problems are revealed in medical investigations. There are various problems and signs, including bloating, constipation, diarrhea, gas, irritable bowel syndrome (IBS), nausea, and poisoning, as well as non-complicated gastroesophageal reflux disease (GERD). In the case of structural GIDs, both abnormal outlook and motility are observed. The structural GIDs involve acute and chronic pancreatitis, various forms of hepatitis, including viral and autoimmune ones, gastric and duodenal peptic ulcer disease and cholelithiasis with their complications, complicated GERD (including Barrett’s esophagus and esophageal strictures), as well as benign and malignant neoplasms, diverticular disease, hemorrhoids, and inflammatory bowel disease (IBD), including Crohn’s disease (CD) and ulcerative colitis (UC) [1,2].

The diagnosis and treatment of GIDs are usually very challenging and require the engagement of a multidisciplinary team including gastroenterologists, radiologists, oncologists, and surgeons. The laboratory, imaging, endoscopic, non-invasive, and invasive investigations are used in the diagnosis of GIDs. Additionally, managing GIDs has a wide spectrum: from observation and conservative treatment to invasive radiological, endoscopic, and surgical procedures. Recently, both the diagnosis and treatment of GIDs have been improved, including, i.a., molecular laboratory tests, gastrointestinal endoscopy with artificial intelligence, targeted and immunological oncological therapy, as well as robotic surgery.

Below are some novel concepts regarding the diagnostics and management of the selected most important current topics related to GIDs, including the renaming of functional GIDs, the role of the human microbiome in GIDs, as well as the diagnosis and treatment of IBD, acute pancreatitis, and pancreatic cancer, are presented.

Recently, communication between the gut and the brain has been widely described [3]. The importance of this relationship was highlighted in 2016 by the Rome Foundation IV diagnostic guidelines, in which functional GIDs were renamed as diseases of gut–brain interactions [3,4]. Numerous bidirectional different routes of communication between the gut and brain have been described. They include direct vagal neural pathways, endocrine factors, and effects of the gut virome [3].

Additionally, many studies on the human microbiome have been published recently. Clinical and population studies have mapped the diversity of the healthy microbiome, as well as its alteration in disease. In vitro and in vivo model system studies have identified and validated pathological mechanisms and clinical targets. Recent methodological advances are important in the introduction of novel treatment strategies in microbiome-related diseases [5].

Changes in the human gut microbiome have been related to various gastrointestinal and liver diseases, including IBD, colorectal cancer (CRC), alcohol-associated liver disease, and nonalcoholic fatty liver disease [6]. Bacteriophages, used against pathogenic bacterial infections, can also precisely modulate the intestinal microbiome and give promising therapeutic effects for numerous gastrointestinal diseases [6].

Regarding diagnostics of IBD, non-invasive, accessible, and cost-efficient biomarkers are needed for the diagnosis of the disease [7]. In recent years, an increasing number of novel serum and fecal biomarkers have been discovered, including serum leucine-rich glycoprotein, anti-*Saccharomyces cerevisiae* antibody (ASCA), perinuclear anti-neutrophil cytoplasmic antibody (pANCA) (with high specificity for IBD), cytokines such as granulocyte colony-stimulating factor related to endoscopically active disease, interleukins (IL) IL-6 and IL-2, circulating noncoding RNAs, cathelicidin, trefoil factor 3, 25-hydroxyvitamin D3, extracellular matrix (ECM) components, and growth factors, as well as fecal myeloperoxidase [7].

Concerning the therapy of IBD, recently, mesenchymal stem/stromal cells (MSCs) have been described as a novel treatment due to their immunoregulatory and pro-survival features. MSCs regulate altered inflammatory responses through the secretion of various anti-inflammatory mediators, such as interleukin 10 (IL-10), transforming growth factor-β (TGFβ), prostaglandin E2 (PGE2), tumor necrosis factor-stimulated gene-6 (TSG-6) [8]. There are several studies regarding other novel treatment methods in patients with IBD, including IL-23p19 antibodies guselkumab and risankizumab in patients with CD, the novel Janus kinase (JAK) inhibitor upadacitinib in patients with moderately to severely active UC, as well as the oral integrin α4 inhibitor carotegrast methyl (AJM300) in mildly to moderately active UC [8,9].

Additionally, new concepts in the pathophysiology, diagnosis, and treatment of acute pancreatitis (AP) and chronic pancreatitis (CP) have been developed recently [10]. The most important findings include novel serum biomarkers—fatty acid ethyl esters (FAEEs)—for the diagnosis of alcohol-related AP, new concepts regarding monocyte/macrophage participation in the immune reaction [10], and the safety and superiority of a low-fat, solid diet introduced immediately upon the decision for admission, regardless of symptoms or laboratory parameters upon conventional nutrition with a progressive diet in relation to the improvement of clinical and laboratory parameters. According to a recent multi-center randomized controlled study by Ramirez-Maldonado [11], early feeding in mild and moderate AP is not only safe but is significantly associated with a lower rate of complications (4.2% vs. 18.3%) and a shorter duration of hospitalization (3.4 days vs. 8.8 days) [10,11]. In addition, the appropriateness of early, aggressive parenteral hydration has been considered [10,12]. de-Madaria et al. [12], in another multi-center randomized-controlled study, reported that early aggressive fluid resuscitation is associated with a higher rate of fluid overload without improvement in clinical outcomes in patients with AP [12]. Generally, it is known that enteral nutrition (EN) is the choice method of feeding in patients with severe AP (SAP) in whom oral feeding is not possible. Early EN (EEN) (<48 h of admission) is better than delayed EN (DEN), and EN should be started within 48 h of admission. Parenteral nutrition is reserved for patients with intolerance or impossibility of EEA [13]. A recent multi-center randomized controlled study by Boxhoorn et al. [13] showed the advantages of the non-operative management of infected pancreatic fluid and the safety of postponing intervention with drainage [10,14]. In addition, patients undergoing a postponed drainage strategy required a lower number of invasive interventions [14].

Pancreatic cancer (PC) is a very aggressive neoplasm characterized by a very poor prognosis (5-year survival rate of 5–8% and a median survival of 5 months). The most common PC type is pancreatic ductal adenocarcinoma (PDAC) noted in 95% of patients [15]. The diagnosis is frequently delayed due to its aggressive biology and late manifestation. This fact is related to a poor prognosis. Recently, the first single-cell and spatial transcriptomic atlases of PDAC were reported, a role of Schwann cells within the tumor microenvironment in perineural invasion via the activation of JUN has been reported, and the role of aerobic exercises in decreasing PDAC growth via the activation of CD8+ T lymphocytes, mediated by cytokines (IL-15–IL-15RA), has been described [16]. Due to a poor prognosis and advanced staging at the time of diagnosis, the early detection of PDAC is an important issue. Progress in the genetic and molecular analysis of various human specimens, involving blood, as well as pancreatic tissue, pancreatic juice, and pancreatic cystic fluid, creates the possibility of early PDAC diagnosis, using molecular approaches. The non-invasive assessment of genetic alterations in human blood allows the early diagnosis of PDAC and precancerous lesions, including mucinous cystic neoplasms (MCNs) and intraductal papillary mucinous neoplasms (IPMNs). Mutations in KRAS and/or GNAS are specifically noted in mucinous cysts, including IPMNs, whereas alterations in CDKN2A, TP53, and/or SMAD4 are specific for advanced tumors [17]. Regarding the identification of precancerous lesions, the diagnosis and management of pancreatic cystic tumors (PCTs), including MCNs and IPMNs, is a very important clinical problem [18]. Clinical decision-making for patients with pancreatic IPMNs is still challenging. The management of branch duct-IPMN (BD-IPMN) is especially controversial. The most important issue is the correct selection of patients who require surgery at the right time, with no unnecessary risk of surgical treatment leading to potential complications related to pancreatectomy [19].

Molecular, genetic, and epigenetic diagnostic tools, novel biomarkers, and promising therapeutic targets are promising in PDAC therapy. The implementation of microarray technology and the application of large omics-based data repositories are important in the diagnosis and treatment of PDAC. Numerous molecular analyses based on RNA interference can be used in novel therapeutic targets for patients with PDAC [20].

In conclusion, there are numerous interesting research areas regarding the novel diagnostic and therapeutic approach for GIDs. This topic is very broad, and it is not possible to discuss all of the essential problems in one short article. Only some recently published issues, the most important topics from the authors’ perspectives, were mentioned above. Therefore, numerous original research and review papers related to all novel aspects of diagnostics and treatment of GIDs regarding laboratory diagnostics as well as the pharmacological, immunological, and targeted treatment of these disorders are invited.
